# Cationic Polymers Remarkably Boost Haloalkane Dehalogenase Activity in Organic Solvent Solutions and the Molecular Implications

**DOI:** 10.3390/molecules28196795

**Published:** 2023-09-25

**Authors:** Yin Wu, Yan Sun

**Affiliations:** 1 Department of Biochemical Engineering, School of Chemical Engineering and Technology, Tianjin University, Tianjin 300350, China; yinwu@tju.edu.cn; 2 Key Laboratory of Systems Bioengineering and Frontiers Science Center for Synthetic Biology (Ministry of Education), Tianjin University, Tianjin 300350, China

**Keywords:** haloalkane dehalogenase, cationic polymer, additives, organic solvents, molecular dynamics simulation, biocatalysis

## Abstract

Applications of haloalkane dehalogenase DhaA in biocatalysis are limited by its unfavorable performance in organic solvents. Our previous work proved that mutations of surface positive-charged residues enhanced the organic solvent resistance of DhaA, which inspired us to explore the effect of cationic polymers on DhaA in organic solvents. Remarkably boosted performance was achieved in different organic solvent solutions by introducing cationic polymers, for example, there was a 6.1-fold activity increase with poly(allylamine hydrochloride) and a 5.5-fold activity increase with poly(ethylene imine) in 40 vol.% dimethylsulfoxide. The presence of cationic polymers protected DhaA from damage by organic solvents and increased the substrate concentration around the enzyme-polymer complex. Fluorescence spectroscopy and molecular dynamics simulations revealed that the binding of cationic polymers onto DhaA weakened the interactions between organic solvents and DhaA, decreased the organic solvent solvation level around DhaA, and enhanced the structural stability of DhaA in organic solvents. This comprehensive understanding of the effect of cationic polymers on DhaA can help to broaden the applications of DhaA in organic solvent-involved biocatalysis.

## 1. Introduction

Halogenated compounds, which are byproducts commonly produced in organic solvent (OS)-based industries, can become pollutants and potentially life-threatening even at an infinitesimal concentration after being released into the environment. For instance, as a byproduct in the synthesis of various chemicals, 1,2,3-trichloropropane exhibits carcinogenic and mutagenic effects, and the California maximum contaminant level of 1,2,3-trichloropropane (0.005 μg·L^−1^) was set by the California State Water Resources Control Board in 2017 based on cancer risk [1]. In addition, hexachlorocyclohexane was found to cause male infertility by suppressing human sperm motility at a very low concentration (less than 500 nM) [2]. As an important member of the α/β-hydrolase superfamily, haloalkane dehalogenases (HLDs) catalyze the hydrolytic cleavage of carbon-halogen bonds in halogenated compounds to generate the corresponding alcohol, a halide ion, and a proton [3,4,5,6]. Thus, HLDs have potential applications in the bioremediation of toxic environmental pollutants, biosensors for environmental pollutants, the decontamination of chemical warfare agents, and protein labeling and cell imaging for protein analysis [7,8,9,10]. However, HLDs exhibit reduced activity, poor stability, and even deactivation in the presence of OS molecules [11,12], which greatly hinders the applications of HLDs in aqueous OS mixtures.

In order to overcome the above problems, numerous approaches have been utilized to improve the catalytic performance of HLDs in OSs. For example, poly(ethylene glycol) (PEG) conjugation was reported to enhance the enzymatic performance of HLDs [13]. The environmental stability of HLDs was strengthened using a self-assembly directed nano-hybrid with iron phosphate [14]. The modification of residues in an access tunnel was observed to greatly improve the stability and resistance of HLDs to OSs [15]. In our previous work, an energy analysis of molecular dynamics (MD) simulations indicated that the positive-charged residues (Arg and Lys) of DhaA (the HLD from *Rhodococcus rhodochrous*) exhibited an attraction to a substrate. In addition, an increase in surface positive-charged residues was shown to have conducive effects on improving the OS resistance of DhaA, especially for a four-substitution variant (E16R/E93R/E121R/E257R), which exhibited a 5-fold improvement in OS resistance (the residual activity ratio after 30 min incubation in the presence and absence of OSs) and a 7-fold half-life increase in 40 vol.% dimethylsulfoxide (DMSO) [16].

Adding polymers into the reaction environment provides a convenient solution for increasing enzymatic performance and is highly desirable in many applications. The presence of polymer additives has been proven to enhance the activity, thermostability, and storage stability of enzymes in aqueous buffer [17,18,19]. For instance, zwitterionic polymers were found to significantly enhance the activity and thermal stability of lipase, lysozyme, and catalase [20]. Poly(γ-glutamic acid) was observed to improve the activity and stability of carbonic anhydrase and α-amylase by up to 48%, and the protease performance was increased by up to 200% in the presence of poly(acrylic acids) (PAAs) [21]. Furthermore, ionic polymers generate a hydrophilic microenvironment around the enzymes and prevent their inactivation from partition of OS molecules [22,23], which greatly improve the enzymatic performance in OS solutions.

Inspired by our previous work [16], this study was designed to systematically explore the effect of cationic polymers on DhaA in OS solutions. A remarkably boosted activity of DhaA was observed in the presence of cationic polymers including poly(allylamine hydrochloride) (PAH) and poly(ethylene imine) (PEI). The influence of cationic polymer concentration on the catalytic activity of DhaA was evaluated, and the kinetic characterization and OS stability of DhaA at specific polymer concentration were detected. Furthermore, different OS solutions and polymer additives (i.e., cationic polymer PEI, anionic polymer PAA, and neutral polymer PEG) were chosen to further explore the effect of polymer additives on DhaA in OS solutions. Fluorescence spectroscopy and MD simulations were utilized to identify the structural and molecular mechanisms underlying the improved performance of DhaA caused by cationic polymers.

## 2. Results and Discussion

### 2.1. Effect of PAH Concentration on Catalytic Performance

**Figure 1:**
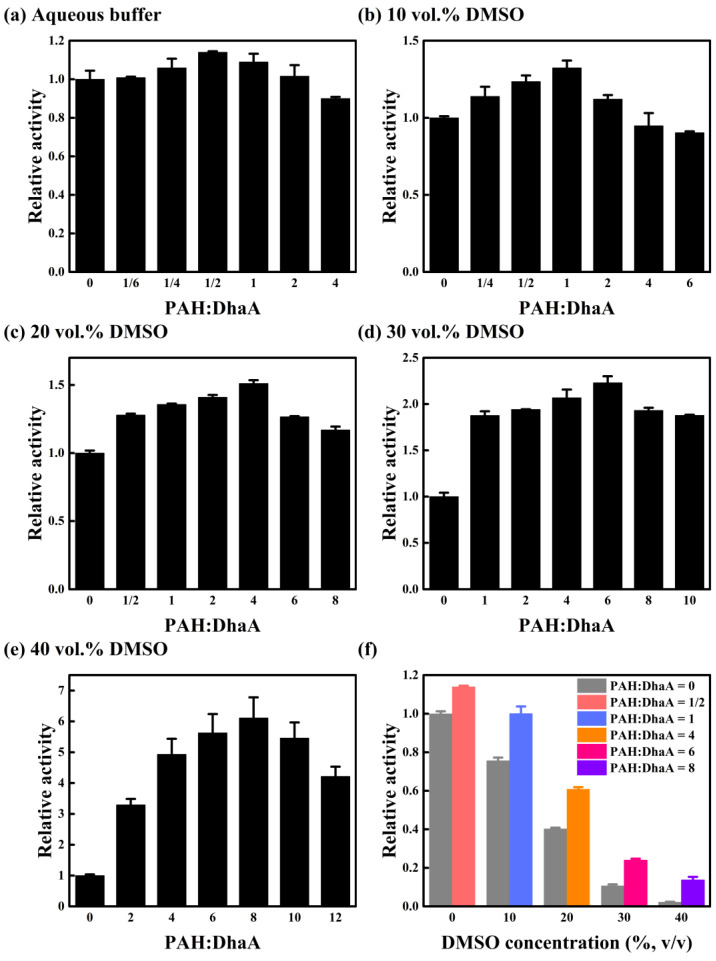
(**a**–**e**) Effect of PAH concentration on the activity of DhaA in different concentrations of DMSO solutions. (**f**) The catalytic activity of DhaA at the optimal molar ratios of PAH to DhaA in different concentrations of DMSO solutions (pH 8.0).

The effect of PAH concentration on the performance of DhaA was explored by detecting the activity of DhaA in the molar ratios of PAH to DhaA ranging from 0 to 12 in different concentrations of DMSO solutions. As shown in Figure 1a–e, the enzyme activity in different DMSO concentrations increased with the increase in PAH concentration until a certain concentration (PAH:DhaA = 1/2 for aqueous buffer, PAH:DhaA = 1 for 10 vol.% DMSO, PAH:DhaA = 4 for 20 vol.% DMSO, PAH:DhaA = 6 for 30 vol.% DMSO, and PAH:DhaA = 8 for 40 vol.% DMSO), and then reduced when the PAH concentration increased further. For poor water-soluble substrates, the presence of DMSO enhanced the solubility of substrates, which was favorable to the enzyme activity. On the other hand, the enzyme activity and stability can be negatively affected by denaturation, conformational rigidity, or inhibition when DMSO concentration increases [11]. Therefore, the activity of pure DhaA decreased with the increase in DMSO concentration, while it was seen that the enzyme activity at the optimal PAH concentration was significantly higher than that of the pure enzyme at each DMSO concentration (Figure 1f). The enzyme with the optimal PAH concentration (PAH:DhaA = 1) in 10 vol.% DMSO showed comparable activity to that of the pure enzyme in the aqueous buffer. In the presence of PAH, the activity in 30 vol.% and 40 vol.% DMSO retained 24% and 14% of the activity of the pure enzyme in the aqueous buffer, respectively. By contrast, the activity of the pure enzyme in 30 vol.% and 40 vol.% DMSO only retained 11% and 2% of that in the aqueous buffer, respectively. It is worth noting that the optimal PAH concentration and the corresponding maximum relative activity increased simultaneously with increasing DMSO concentration. For instance, the optimal molar ratio of PAH to DhaA in an aqueous buffer (1:2) generated a 14% improvement in DhaA activity (Figure 1a), while in 20 vol.% DMSO, the optimal molar ratio increased to 4:1 with a 51% improvement in activity (Figure 1c). The optimal molar ratio further enlarged to 8:1, and resulted in a 6.1-fold activity increase in 40 vol.% DMSO (Figure 1e).

Fluorescence spectroscopy was used to explore the conformational transitions of DhaA in OS solutions (Figure S2), and the maximum fluorescence emission wavelength (λ_max_) values of DhaA at different PAH concentrations are summarized in Table S1. The λ_max_ values of DhaA in the aqueous buffer exhibited an inappreciable change after adding different concentrations of PAH, indicating that using PAH as an additive has little influence on the tertiary structure of DhaA in the aqueous buffer. However, the λ_max_ values of DhaA showed significant blue shifts in 40 vol.% DMSO in the presence of PAH, and the degree of blue shift enlarged with increasing PAH concentration, demonstrating that the presence of PAH greatly improved the structural stability of DhaA in 40 vol.% DMSO. In addition, the positive effect on enzyme stability increased with the increase in PAH concentration, which was consistent with the activity assays (Figure 1).

The above findings revealed that the cationic polymer PAH had complicated effects on the catalytic performance of DhaA. The negative charge of DhaA at the reaction condition and the positive charge of PAH drove the binding of enzyme and polymer by electrostatic interactions. The turbid system was observed after mixing DhaA and PAH, and the phenomenon became notable with the increase of PAH concentration, indicating the formation of enzyme/polymer aggregates, which could be regarded as a kind of carrier-free immobilized enzyme. In addition, it has been reported that under the influence of ionic polymers, the concentrations of OSs, oxygen, and hydrophobic molecules reduced in the enzyme environment [22,23]. This could be favorable for the enzyme stabilization. Moreover, the coated enzyme could be protected from interaction with gas bubbles [24], thus increasing the enzyme stability in practical applications. On the other hand, PAH binding on enzyme molecules would also hinder the substrate’s access to the active site of DhaA, which was averse to the enzyme activity. The binding free energy contribution of each residue in DhaA to the substrate binding calculated using MD simulations in our previous work demonstrated that the positive-charged groups on DhaA had an attraction to the substrate [16]. This suggests that there might be also interactions between the positively charged PAH and the substrate molecule, leading to the enrichment of the substrate around the enzyme-polymer complex, which was favorable for increasing enzyme activity. As shown in Figure S3, the solution viscosities slightly increased with the increase in PAH concentration, and the addition of PAH might have hindered the diffusion of both the substrate and enzyme, which had a negative effect on the catalytic activity [25,26]. At low PAH concentrations, the positive effect of PAH played a dominant role, so the activity of DhaA increased with increasing PAH concentration. However, the positive effect was overwhelmed by the negative effect at high PAH concentrations, resulting in a decrease in DhaA activity when PAH concentration further increased. Furthermore, higher DMSO concentration caused greater damage to the enzyme molecule, requiring higher PAH concentration to resist the negative influence of OS molecules. Therefore, the optimal molar ratio of PAH to DhaA increased with increasing DMSO concentration (Figure 1).

### 2.2. Enzymatic Kinetics and Stability in OS Solution

Two specific molar ratios of PAH to DhaA (4:1 and 8:1) were selected to further study the effect of PAH on the enzymatic performance of DhaA. The kinetics of enzymatic reactions in the absence/presence of PAH in 40 vol.% DMSO are shown in Figure S4, and the kinetic parameters are listed in Table 1. A significantly increased *k*_cat_ value and a somewhat increased *K*_m_ value were observed for DhaA in the presence of PAH, representing the remarkably improved catalytic activity and somewhat weakened enzyme-substrate affinity. It is considered that the enhanced catalytic activity of DhaA was attributed to the protection of PAH, which weakened the negative influence of OS molecules on DhaA. Furthermore, the attraction between PAH and the substrate molecule gave rise to the enrichment of the substrate around the enzyme-polymer complex, which was also favorable in increasing the *k*_cat_ value. On the other hand, the PAH binding on enzyme molecule also hindered the substrate’s access to the active site, weakening the enzyme-substrate affinity. However, due to the significant increase in *k*_cat_, the catalytic efficiency (*k*_cat_/*K*_m_) at the two PAH/DhaA molar ratios was 4.8 and 5.6 times higher than that of the pure enzyme in 40 vol.% DMSO, respectively (Table 1).

The secondary structure changes of DhaA in the absence/presence of PAH in aqueous buffer and OS solution were explored by examining the circular dichroism (CD) spectroscopy (Figure S5). In the aqueous buffer, the far-UV CD spectroscopy of DhaA in the presence of PAH was similar to that of the pure enzyme, indicating that adding PAH did not influence the secondary structure of DhaA. The high absorbance of DMSO in the far-UV spectral region strongly interfered with the protein signal [27,28], which hindered the detection of secondary structures using CD spectroscopy with adequate accuracy in the DMSO solution. Despite DMSO interference, the spectra above 220 nm of the pure enzyme showed more significant change than that of DhaA with PAH, verifying that the presence of PAH protected the secondary structure of DhaA from the negative effect of DMSO, which was favorable for increasing the enzyme activity in OS solutions (Table 1).

The DhaA stability in OS in the presence of PAH was determined in 40 vol.% DMSO (Figure S6). The inactivation of the enzyme occurred with incubation time, and the residual activity of DhaA with different PAH concentrations was reduced to less than 35% of the original activity after 90 min incubation (Figure S6a). The half-life values in 40 vol.% DMSO of the enzyme with different concentrations of PAH predicted from the fitting are listed in Table S2. As shown in the table, the increase in PAH concentration reduced the half-life of DhaA in 40 vol.% DMSO. The results suggest that compared with the pure enzyme, the enzyme under the protection of PAH was more sensitive to OS molecules, and the intrusion of OS molecules caused greater damage to the catalytic activity, which resulted in a shorter half-life in 40 vol.% DMSO. The fluorescence spectroscopic measurement showed that the difference between the λ_max_ values of DhaA in the presence/absence of PAH in 40 vol.% DMSO greatly reduced after 90 min of incubation (Figure S7), indicating a reduced structural stability difference between DhaA with/without PAH. Despite the shorter half-life of DhaA in the presence of PAH, the residual activity of DhaA in the presence of PAH was far higher than that of DhaA in the absence of PAH during the whole incubation (Figure S6b). This is considered to be due to the much higher enzymatic activity maintained by PAH at the two concentrations. Based on the data shown in Figure S6b, the overall catalytic performance of the enzyme at the two PAH/DhaA molar ratios was calculated to be 4.8 and 4.3 times higher than that of the pure enzyme, respectively, indicating the large benefits achieved by adding PAH to the OS solution.

### 2.3. Enzymatic Performance and Conformational Change in Various OS Solutions

**Figure 2:**
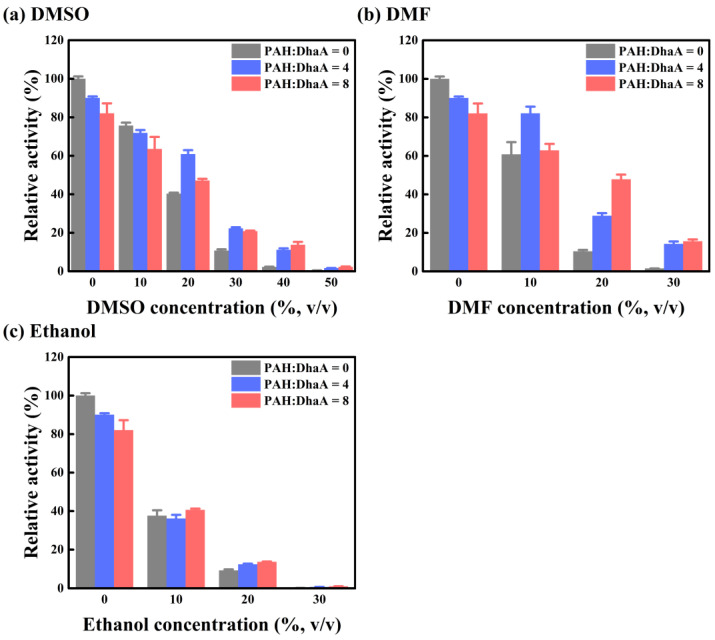
The catalytic activity of DhaA with/without adding PAH in different concentrations of OS solutions, defining the activity of pure enzyme (no PAH) in aqueous buffer as 100%.

The effect of PAH on the performance of DhaA in different OS solutions (DMSO, N,N-dimethylformamide (DMF), and ethanol) was studied, and the results are summarized in Figure 2 and Figure S8. Although ethanol could be considered an HLDs product, as far as we know, there is no relevant research about the product inhibition of DhaA. Therefore, ethanol was only considered as an organic solvent to discuss its influence on DhaA in this study. Because of the two relatively high PAH/DhaA molar ratios used in this Section, the negative effect of PAH (like hindering the accessibility of substrate to the active site of DhaA) played a dominant role at low OS concentrations, leading to a decrease in enzyme activity. Then, the negative effect was overwhelmed by the positive effect (mainly to protect DhaA from the damage of OS molecules) at high OS concentrations, leading to a dramatically enhanced activity of DhaA. For instance, in 30 vol.% DMSO, 20 vol.% DMF and ethanol, the activity of the pure enzyme was only about 10% of that in the aqueous buffer. However, after adding PAH, the activity of DhaA in 30 vol.% DMSO increased by about 100% as compared with that without PAH (Figure 2a). At 20 vol.% DMF, the activity of DhaA at the two PAH/DhaA molar ratios increased by 178% and 360% as compared with that of the pure enzyme, respectively (Figure 2b). In the presence of PAH, the enzyme activity in 20 vol.% ethanol increased by 41% compared with that without PAH (Figure 2c). Furthermore, DhaA at the two PAH/DhaA molar ratios also exhibited enhanced OS resistance (especially in the high-concentration OS solutions) as compared with the pure enzyme (Figure S8).

**Figure 3:**
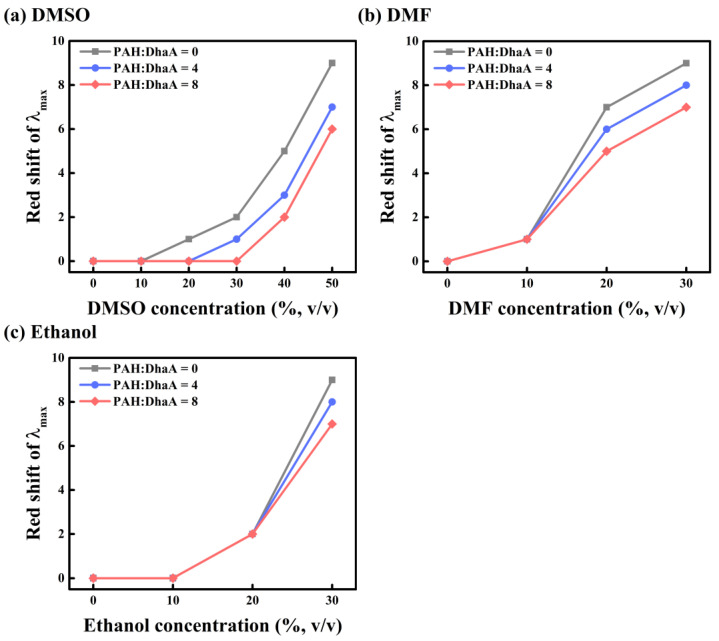
The red shift in λ_max_ of DhaA with/without adding PAH at different concentrations of OS solutions.

The fluorescence spectroscopy of DhaA in the absence/presence of PAH was examined with increasing concentrations of OSs to study the effect of PAH on the tertiary structure of DhaA in different OS solutions (Figure 3 and Figures S9–S11). The λ_max_ of DhaA showed a red-shift with increasing concentrations of OS, demonstrating that the overall structure of DhaA became unstable under the influence of OS molecules [29]. However, the degree of the red-shift greatly weakened after adding PAH to the solutions (Figure 3), indicating the suppression of conformational change by PAH. The above results showed that PAH was able to resist the negative influence of different OS molecules on the enzymatic activity and structural stability of DhaA.

### 2.4. Effects of Polymer Additives with Different Charges

**Figure 4:**
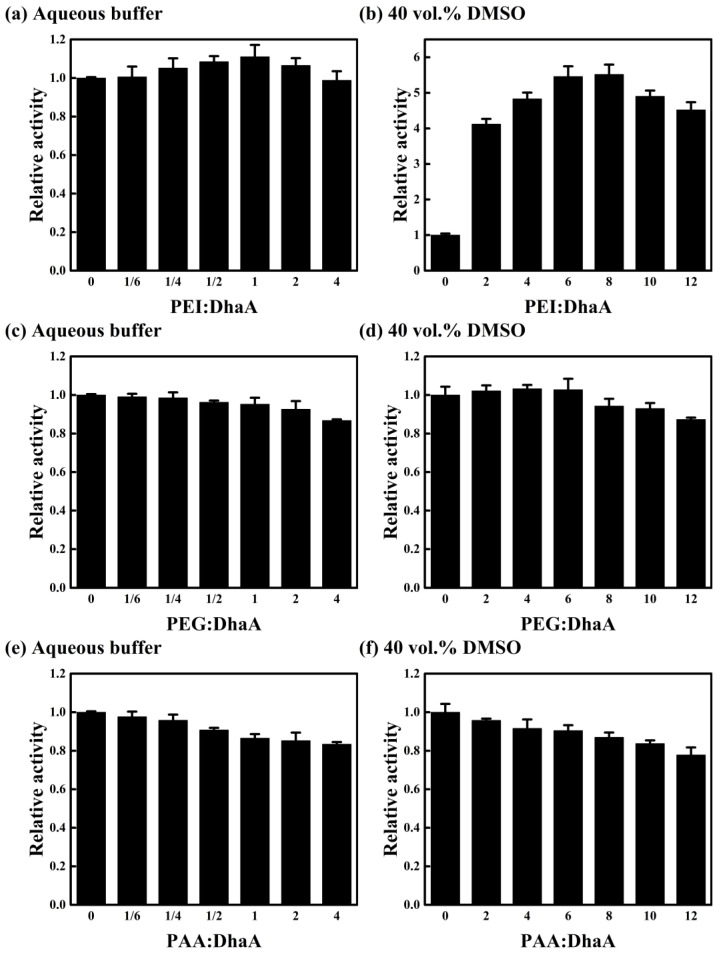
Effect of polymer concentration on DhaA activity in the aqueous buffer and 40 vol.% DMSO: (**a**,**b**) cationic polymer PEI, (**c**,**d**) neutral polymer PEG, and (**e**,**f**) anionic polymer PAA.

The influence of polymer charge was studied using a comparison of three different polymer additives (cationic polymer PEI, anionic polymer PAA, and neutral polymer PEG) in the aqueous buffer and 40 vol.% DMSO (Figure 4). As shown in Figure 4a,b, the activity of DhaA in the aqueous buffer and 40 vol.% DMSO increased first and then decreased with PEI concentration, and the optimal PEI concentration and the corresponding maximum relative activity were found to increase simultaneously with DMSO concentration. These phenomena were consistent with the earlier findings for PAH (Figure 1a,e). However, an increasing PEG/PAA concentration caused a decrease in DhaA activity in the aqueous buffer and 40 vol.% DMSO (Figure 4c–f).

Fluorescence spectroscopy was used to explore the effects of different polymer additives on the tertiary structure of DhaA (Figure S12). It was observed that DhaA exhibited similar λ_max_ values in the presence of different polymer additives (Figure S12a,b), so the difference in polymer additives had an inappreciable influence on the tertiary structure of DhaA in the aqueous buffer. However, compared with PEG and PAA, the λ_max_ of DhaA in the presence of PEI showed a blue-shift in 40 vol.% DMSO, and the degree of blue-shift increased with increasing PEI concentration (Figure S12c,d). These results showed that the cationic polymer PEI had a positive effect on the structural stability of DhaA in OS solution, and increasing PEI concentration strengthened the conducive effect, which was consistent with the effect of PAH (Figure S2b).

The similar charge properties of PAH and PEI drove the formation of similar enzyme/polymer aggregates, i.e., a kind of carrier-free immobilized enzyme, and resulted in the similar favorable effect on the enzymatic performance in organic media, both in the protection on DhaA and the attraction with substrate. By contrast, PEG and PAA did not exhibit the beneficial effects on the enzyme that were observed for PAH and PEI. The neutral polymer PEG could not form a stable enzyme-polymer complex with DhaA. For PAA, its negative charge was similar to that of DhaA, and the anionic polymer at the reaction condition hindered the formation of an enzyme-polymer complex. These made PEG and PAA exhibit little effect in protecting DhaA from damage caused by OS molecules (Figure S12c,d). Furthermore, as compared with the cationic polymers PAH and PEI, there was no attraction between the substrate and PEG/PAA owing to their charge properties. Instead, increasing the concentrations of the two polymers led to an increase in solution viscosities (Figure S3), and the polymers might have hindered the diffusion of both the substrate and enzyme [25,26], consequently causing a decrease in the enzymatic activity (Figure 4c–f).

**Figure 5:**
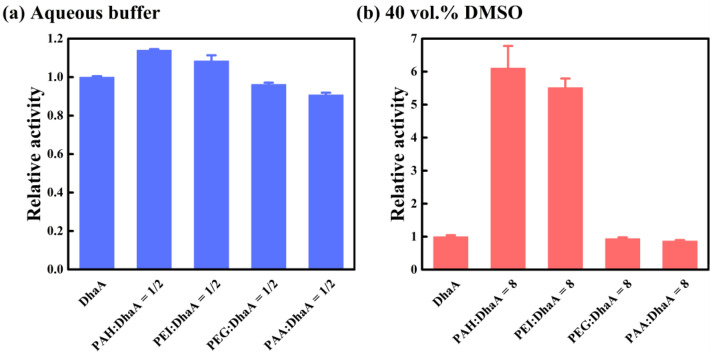
Catalytic activities of DhaA at specific polymer concentrations.

The effects of different polymer additives (cationic polymers PAH and PEI, anionic polymer PAA, and neutral polymer PEG) on the activity of DhaA in the aqueous buffer and 40 vol.% DMSO are summarized in Figure 5. In the aqueous buffer, the presence of PAH and PEI caused an over 10% improvement in the activity of DhaA (Figure 5a). However, the addition of PEG and PAA resulted in a decrease in DhaA activity. Furthermore, PAH and PEI exhibited remarkably favorable effects on the activity of DhaA in 40 vol.% DMSO, which resulted in an over 5.5-fold activity increase, while the negative effects of PEG and PAA caused a reduced activity of DhaA in OS solutions (Figure 5b). These results showed that in contrast to the neutral polymer PEG and the anionic polymer PAA, the cationic polymers PAH and PEI remarkably enhanced the catalytic activity and structural stability of DhaA (especially in the high-concentration OS solutions).

### 2.5. Molecular Mechanism Underlying the Improved Enzymatic Performance

MD simulations generate a complementary approach to explore the connection between protein dynamics and the stability of enzymes in OSs, which has been shown to exhibit high consistency with numerous experimental measurements [30,31]. MD simulations for DhaA in the absence/presence of PAH in 40 vol.% DMSO were thus performed to identify the molecular mechanism underlying the improved enzymatic performance. As can be seen in Figure S13, there was no obvious difference in the root mean square deviation (RMSD) and radius of gyration (R_g_) values between DhaA in the absence and presence of PAH. Furthermore, the similar time-averaged total, hydrophobic, and hydrophilic solvent accessible surface area (SASA) values for DhaA were observed in the absence/presence of PAH (Figure S14), suggesting that PAH did not appreciably affect the overall exposure degree of DhaA.

**Figure 6:**
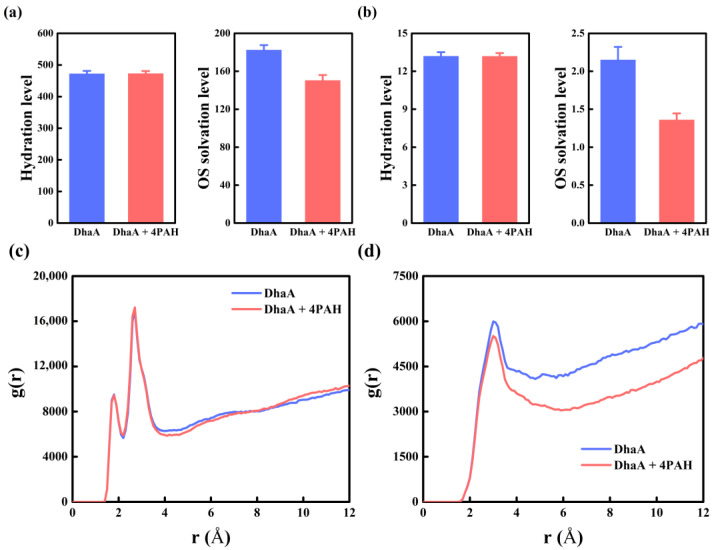
Hydration level and OS solvation level around (**a**) the enzyme and (**b**) the catalytic triad in the absence/presence of PAH in 40 vol.% DMSO averaged over the last 40 ns of MD trajectories. Water molecules whose O atom was within a 3.5 Å distance cut-off of any non-hydrogen atom of the enzyme were described as the first hydration shell and the number of water molecules as the hydration level. DMSO molecules whose S2 atom was within a 6.8 Å distance cut-off of any non-hydrogen atom of the enzyme were described as the OS layer and the number of DMSO as the OS solvation level. The RDF of (**c**) water molecules and (**d**) DMSO molecules around the enzyme in the absence/presence of PAH in 40 vol.% DMSO. RDF was calculated over the last 40 ns from three independent MD simulation runs.

The solvation properties were then examined in the absence/presence of PAH, including the hydration level around the enzyme, the OS solvation level around the enzyme, the hydration level around the catalytic triad, and the OS solvation level around the catalytic triad (Figure 6a,b). The hydration level around the enzyme and the catalytic triad exhibited inappreciable changes in the absence/presence of PAH. The radial distribution function (RDF) of water molecules around the pure enzyme was similar to that in the presence of PAH (Figure 6c), indicating the inappreciable influence of PAH on the hydration degree of DhaA. On the other hand, decreased OS solvation levels around the enzyme and the catalytic triad were observed in the presence of PAH, and the density of DMSO molecules around DhaA in the presence of PAH was much lower than that of the pure enzyme based on the analysis of the RDF of DMSO molecules (Figure 6d). As reported previously, the hydration level maintenance and the OS solvation level reduction weakened the adverse contacts between OS molecules and the enzyme, consequently decreasing the negative effect of OS molecules [32].

The average non-bond binding energy (ΔG_non-bond_) between water/DMSO molecules and the overall structure of the enzyme were analyzed to evaluate the interactions between water/DMSO molecules and DhaA in the absence/presence of PAH, and the results are listed in Tables S3 and S4. The presence of PAH greatly reduced the ΔG_non-bond_ values between water/DMSO molecules and DhaA, which might be attributed to the fact that PAH binding on enzyme molecule decreased the contacts between water/DMSO molecules and DhaA. As a hydrophilic polymer, PAH exhibited a 5.7 times higher ΔG_non-bond_ value with water molecules than that of PAH with DMSO molecules (Table S5). Although the presence of PAH weakened the interaction between DhaA and water molecules, the stronger interaction between PAH and water molecules finally resulted in an inappreciable change in the hydration level around DhaA. On the other hand, the decreased interaction between DhaA and DMSO molecules caused by PAH and the weak interaction between PAH and DMSO molecules together gave rise to a remarkable decrease in the OS solvation level around DhaA (Figure 6).

**Figure 7:**
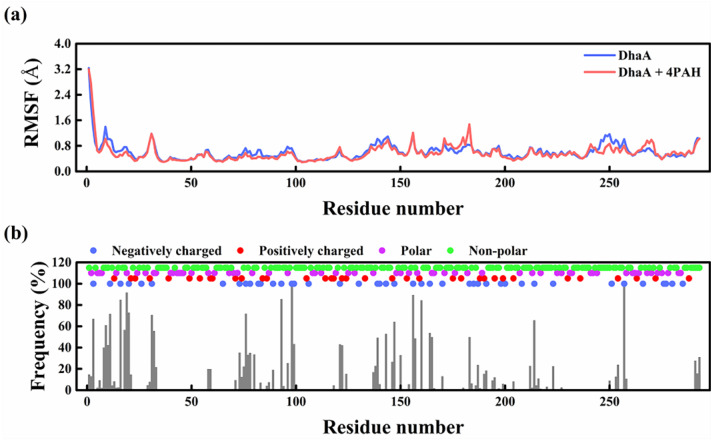
(**a**) RMSF of the Cα atoms per residue analysis of the enzyme in the absence/presence of PAH using MD simulations. (**b**) Time-averaged frequency of contacts between the residues of DhaA and PAH chains in 40 vol.% DMSO. Residue-PAH contact was defined as a residue-PAH distance of 3.5 Å or less. Contact frequency was calculated over the last 40 ns from three independent MD simulation runs.

The root mean square fluctuation (RMSF) in DhaA residues in the presence of PAH showed reduced values in several regions as compared with the pure enzyme (Figure 7a), and the regions were consistent with those of DhaA residues with high PAH contact frequencies (Figure 7b). The decreased local flexibility of the enzyme was reported to have a positive effect on the stability of the enzyme in OSs [32]. Moreover, hydrogen bonding between DhaA and PAH chains was observed within the simulation time (Figure S15). These results indicate that PAH increased the structural stability of DhaA in OS solutions. Furthermore, the electrostatic attraction of the oppositely charged DhaA and PAH at the reaction condition played a dominant role in the interaction between DhaA and PAH (Table S6), so the high PAH contact frequency was mainly located in the regions with a dense distribution of negative-charged residues (Figure 7b).

In summary, the electrostatic interactions between DhaA and PAH drove the formation of enzyme/polymer aggregates, and achieved the carrier-free immobilization of the enzyme. Enzyme immobilization may exhibit many advantages, such as improved enzyme stabilization, increased activity, selectivity, or specificity, and enhanced resistance to chemicals [33,34,35]. In this work, enzyme immobilization was proved to be favorable for enzyme performance in organic media. Specifically, the PAH binding on enzyme molecule enhanced the structural stability of DhaA in OS solutions, and weakened the adverse contacts and interactions between OS molecules and DhaA, resulting in the decreased OS solvation level around DhaA. Besides, the hydrophilicity of PAH maintained the hydration level around DhaA. Although the protection effect caused by enzyme immobilization gradually decreased with the increased incubation time owing to the intrusion of OS molecules, the immobilized enzyme still exhibited remarkably enhanced overall catalytic performance compared with the free enzyme in organic media.

## 3. Materials and Methods

### 3.1. Materials

PAH (average M_W_ ~17,500 Da) was purchased from Sigma-Aldrich (St. Louis, MO, USA). PEI (average M_W_ ~10,000 Da) was obtained from Aladdin (Shanghai, China). PEG (average M_W_ ~8000 Da) was purchased from Dingguo Biotech (Tianjin, China). PAA (average M_W_ ~5000 Da) was obtained from Rhawn Reagents (Shanghai, China), and 4-bromomethyl-6,7-dimethoxycoumarin was purchased from Jiuding Chemistry (Shanghai, China). DMSO was obtained from Heowns (Tianjin, China). DMF and ethanol were obtained from Jiangtian Chemical Technology (Tianjin, China). All the other chemical reagents used in this study were of analytical grade.

### 3.2. Enzyme Preparation

DhaA was expressed and purified following the protocol reported earlier [11]. Briefly, the expression strain *E. coli* BL21(DE3) was grown at 37 °C to an optical density (OD_600_) of 0.6–0.8 in Luria-Bertani medium (200 mL) containing kanamycin (50 μg·mL^−1^). Protein overexpression was induced by adding isopropyl β-D-1-thiogalactopyranoside to a final concentration of 0.5 mM and the culture was continued for 18 h at 16 °C. The cells were harvested using centrifugation at 5000× *g* for 30 min at 4 °C, and the cell pellet was disrupted using ultrasonication in lysis buffer (20 mM HEPES-NaOH, 500 mM NaCl, 10 mM imidazole, pH 7.5). Centrifugation of the cell homogenate was performed at 10,000× *g* for 30 min at 4 °C to remove cell debris, followed by filtration through a 0.45 μm syringe filter. DhaA was purified from the clarified lysate in an AKTA Basic chromatography system (GE Healthcare, Marlborough, MA, USA) using a 5 mL affinity chromatography column filled with Ni-NTA Sepharose resin. The column was washed using lysis buffer to remove unbound and weakly bound proteins, and finally eluted using elution buffer (20 mM HEPES-NaOH, 500 mM NaCl, 300 mM imidazole, pH 7.5) to recover the purified enzyme fraction. The recovered protein fraction was further purified in an AKTA Basic chromatography system (GE Healthcare, Marlborough, MA, USA) using a Superdex 200 Increase 10/300 GL size-exclusion chromatography column against desalting buffer (50 mM HEPES-NaOH, pH 8.0). The purity was evaluated using sodium dodecyl sulfate-polyacrylamide gel electrophoresis, and the concentrations were determined with BCA Protein Assay Kit (Solarbio, Beijing, China).

### 3.3. Enzymatic Assays

The activity of DhaA in the presence/absence of a polymer additive was determined using 4-bromomethyl-6,7-dimethoxycoumarin as substrate in a microplate reader (Tecan, Infinite M200 Pro, Männedorf, Switzerland) using a previously reported method with slight modification [36]. As a hydrophobic substrate with poor water solubility, 4-bromomethyl-6,7-dimethoxycoumarin was first dissolved in DMSO before being added to the reaction system. Then, 1 μL freshly prepared substrate solution (final concentration, 100 μM) was added into 200 μL HEPES buffer (50 mM, pH 8.0) or OS solutions containing 4 μg·mL^−1^ enzyme at 30 °C, and fluorescence intensity was measured from the top using excitation/emission monochromators set to wavelengths of 345/437 nm. Before the measurement, the microtiter plate was shaken for 2 s and then the increase in fluorescence intensity was monitored at regular time intervals. To obtain a series of polymer/DhaA molar ratios used in this study, the mass concentrations of DhaA and polymers, the molecular mass of DhaA, and the average molecular weights of polymers were utilized to calculate the corresponding molar concentrations of DhaA and polymers. Activity assays were performed at least in triplicate.

The enzymatic kinetics of DhaA in the presence/absence of a polymer additive was determined by measuring the initial catalytic reaction rate at different substrate concentrations. The concentration of enzyme was kept at 4 μg·mL^−1^. Michaelis constant (*K*_m_) and the maximum reaction rate (*V*_max_) were determined by fitting the measured reaction rates to the Michaelis-Menten equation (Equation (1)) using OriginPro b9.3 software.

(1) $$v=\frac{V_{\max}[S]}{K_{m}+[S]}$$

where *v* is the reaction rate (μM·s^−1^), [*S*] is the substrate concentration (μM), *V*_max_ is the maximum rate (μM·s^−1^), and *K*_m_ is the Michaelis constant (μM).

The pure DhaA protein and the mixture of DhaA and polymer additive were incubated in 40 vol.% DMSO at 30 °C to monitor the kinetics of OS inactivation, and the residual activity was detected in the reaction system containing 40 vol.% DMSO. Half-life (min) was defined as the time required for an enzyme to reach the residual activity of one-half of its initial value. The one-step inactivation dynamics model was previously reported to calculate the half-life of DhaA in 40 vol.% DMSO [15], and thus it was used in this work to fit the inactivation process of the DhaA protein in the presence/absence of a polymer additive. The half-life (*T*_1/2_, min) was calculated according to the following equation,

(2) $$T_{1/2}=\frac{\ln2}{k_{d}}$$

where *k_d_* is the first-order deactivation rate constant (min^−1^) described by Equation (3),

(3) $$-\frac{dE}{dt}=k_{d}E$$

where *E* is the residual enzyme activity (μmol·min^−1^) and *t* is the incubation time (min).

### 3.4. Viscosity Assays

The kinematic viscosities at different concentrations of various polymer additives were measured using a normal-flow U-tube capillary viscometer (Qihang Glass, Shanghai, China) in a thermostat water bath (30 °C). The viscometer constant was 0.003376 mm^2^·s^−2^. Viscosity assays were performed at least in triplicate.

### 3.5. Fluorescence and CD Spectroscopy

The fluorescence spectroscopy of DhaA in the presence/absence of polymer additives was measured to investigate the influence of OS molecules on the conformational change. The fluorescence excitation setting varies with different proteins, for instance, 270 nm for *Candida rugosa* lipase [20], 280 nm for polyethylene terephthalate hydrolase [37], and 295 nm for *Mycobacterium leprae* heat shock protein 18 [38,39]. In this study, the fluorescence excitation was set at 270 nm according to the detection result (Figure S1). Samples were loaded into a quartz cuvette with a path length of 1.0 cm, which was excited at 270 nm for fluorescence spectroscopy experiments on a Luminescence Spectrometer (PerkinElmer, Waltham, MA, USA). The emission spectra were monitored in the range of 290–420 nm at a scanning speed of 200 nm·min^−1^. The slit widths were 5.0 nm for both excitations and emissions. Emission baseline corrections were performed with a blank buffer scan. The reported spectra represented the average of three accumulations subtracted from the corresponding background fluorescence.

The far-UV CD spectra were detected using a J-810 CD spectrometer (JASCO Corporation, Tokyo, Japan) using a 1 mm path length cuvette. The scanning was conducted over the wavelength ranging from 190 to 260 nm in aqueous buffer and OS solution with a 1 s response time and a 2 nm bandwidth at a scanning speed of 100 nm·min^−1^, and ellipticity data were collected. The averaged spectra of three scans were reported, from which the spectrum of a buffer blank was subtracted.

### 3.6. Molecular Dynamics Simulation

All simulations were performed using GROMACS 5.1.4 simulation package [40,41] and parameters from Amber99SB*-ILDN force field [42] for DhaA. The restrained electrostatic potential charges of DMSO and PAH were assigned using Multiwfn 3.7 [43], and the force fields were constructed for them. The crystal structure of DhaA was obtained from the Protein Data Bank (PDB ID 4HZG) [44], and DhaA was solvated into a box of SPC water molecules. PAH chains were randomly added into the system when calculating the system of enzyme-polymer complexes. DMSO molecules with a volume fraction of 40% were then added to the box, and the remaining space was filled with water molecules. Cl^−^ was used to neutralize the total net charge of the systems. The energy minimization of every system was accomplished with 50,000 steps of steepest descent, and the systems were heated to 303 K for 100 ps with velocity-rescale under a constant amount of substance (N), volume (V), and temperature (T) (NVT ensemble). Then, the systems were equilibrated for 100 ps at 303 K and 1 atm using a Parrinello-Rahman pressure coupling (NPT ensemble). After proper minimizations and equilibrations, the final MD simulations were conducted in the NPT ensemble for 100 ns. Electrostatic interaction was disposed of using a particle-mesh Ewald algorithm, which was also utilized to describe long-range electrostatics. A 2.0 fs timestep was used alongside the Verlet algorithm. The simulations were performed with a 1.4 nm cutoff of the neighboring atom list, Coulomb potential energies, and Lennard-Jones potential. VMD 1.9.3 was applied for the visualization and analysis [45].

## 4. Conclusions

In this work, we systematically explored the complicated effect of cationic polymers on the enzymatic performance of DhaA in OS solutions. Compared with neutral and anionic polymers (PEG and PAA), the 6.1-fold activity increase and 5.6-fold improvement in catalytic efficiency in 40 vol.% DMSO were observed in the presence of cationic polymers including PAH and PEI, and the enhancement caused by cationic polymers gradually weakened with the increase of incubation time. The opposite charges of DhaA and the cationic polymers at the reaction condition drove the formation of enzyme/polymer aggregates, which could be regarded as a kind of carrier-free immobilized enzyme. This protected DhaA from the negative influence of OS molecules including DMSO, DMF, and ethanol. Furthermore, the attraction between the cationic polymers and the substrate molecule enriched the substrate around the enzyme/polymer aggregates. Fluorescence spectroscopy and MD simulations revealed the protection mechanisms underlying the effect of cationic polymers on the enzyme molecule in OS solutions. The systematical understanding of the effect of cationic polymers on DhaA can help to broaden practical applications of DhaA in OSs.

## Figures and Tables

**Table 1:** Kinetic parameters of DhaA with different concentrations of PAH in 40 vol.% DMSO.

PAH:DhaA	*K*_m_ (μM)	*k*_cat_ × 10^3^ (s^−1^)	*k*_cat_/*K*_m_ × 10^3^ (s^−1^μM^−1^)
0:1	75.7 ± 5.2	16 ± 1	0.21 ± 0.01
4:1	83.9 ± 8.3	84 ± 5	1.00 ± 0.05
8:1	91.0 ± 7.3	107 ± 5	1.17 ± 0.05

## Data Availability

Data are available upon request from the authors.

## Supplementary Materials

The following supporting information can be downloaded at: https://www.mdpi.com/article/10.3390/molecules28196795/s1, Table S1: The λ_max_ of DhaA in the presence of different PAH concentrations in the aqueous buffer and 40 vol.% DMSO solution; Table S2: Comparison of the half-life in 40 vol.% DMSO for DhaA with different concentrations of PAH; Table S3: The averaged non-bond binding energy between water molecules and the overall structure of the enzyme DhaA in the absence/presence of PAH; Table S4: The averaged non-bond binding energy between DMSO molecules and the overall structure of the enzyme DhaA in the absence/presence of PAH; Table S5: The averaged non-bond binding energy between water/DMSO molecules and PAH chains; Table S6: The averaged non-bond binding energy between PAH chains and the overall structure of enzyme DhaA; Figure S1: The contour fluorescence spectra of DhaA; Figure S2: Fluorescence spectra of DhaA in the presence of different concentrations of PAH in the aqueous buffer and 40 vol.% DMSO solution; Figure S3: The kinematic viscosities of the aqueous buffer and 40 vol.% DMSO solution containing different concentrations of various polymer additives; Figure S4: The catalytic rate of 4-bromomethyl-6,7-dimethoxycoumarin by DhaA at different PAH concentrations in 40 vol.% DMSO as a function of substrate concentration; Figure S5: CD spectra of DhaA in the absence/presence of PAH in the aqueous buffer and 40 vol.% DMSO; Figure S6: The residual activity of DhaA at different PAH concentrations in 40 vol.% DMSO at 30 °C as a function of incubation time; Figure S7: Fluorescence spectra of DhaA with different PAH concentrations in 40 vol.% DMSO solution before and after 90 min of incubation; Figure S8: The catalytic activity of DhaA with/without adding PAH in different concentrations of DMSO, DMF, and ethanol solutions; Figure S9: Fluorescence spectra of DhaA with/without adding PAH at different DMSO concentrations; Figure S10: Fluorescence spectra of DhaA with/without adding PAH at different DMF concentrations; Figure S11: Fluorescence spectra of DhaA with/without adding PAH at different ethanol concentrations; Figure S12: Fluorescence spectra of DhaA with different polymer additives in aqueous buffer and 40 vol.% DMSO; Figure S13: RMSD of the Cα atoms and the time course of Rg analysis of the enzyme in the absence/presence of PAH using MD simulations; Figure S14: The time-averaged total, hydrophobic, and hydrophilic SASA of the enzyme in the absence/presence of PAH in 40 vol.% DMSO; Figure S15: The number of hydrogen bonds between enzyme DhaA and PAH chains as a function of simulation time.

## Sample Availability

Samples of the compounds are available from the authors.
