# Supplementary material for: Cationic Polymers Remarkably Boost Haloalkane Dehalogenase Activity in Organic Solvent Solutions and the Molecular Implications
Source: Molecules. 2023 Sep 25;28(19):6795. doi: 10.3390/molecules28196795 (PMC10574148; doi:10.3390/molecules28196795)
Supplement: Supplementary file 1 [file molecules-28-06795-s001.zip › molecules-2590400-supplementary.docx]

**Supporting Information**

**Cationic polymers remarkably boost haloalkane dehalogenase activity in organic solvent solutions and the molecular implications**

Yin Wu ^1^ and Yan Sun ^1,2,^*

^1^ *Department of Biochemical Engineering, School of Chemical Engineering and Technology, Tianjin University, Tianjin 300350, China*

^2^ *Key Laboratory of Systems Bioengineering and Frontiers Science Center for Synthetic Biology (Ministry of Education), Tianjin University, Tianjin 300350, China*

* Corresponding author:

E-mail address: ysun@tju.edu.cn (Y. Sun)

**Table S1.** The maximum fluorescence emission wavelength (λ_max_) of DhaA in the presence of different poly(allylamine hydrochloride) (PAH) concentrations in the aqueous buffer and 40 vol.% dimethylsulfoxide (DMSO) solution.

| **PAH:DhaA** | **λ_max, aqueous buffer_ (nm)** | **λ_max, 40 vol.% DMSO_ (nm)** |
| --- | --- | --- |
| 0:1 | 326 | 331 |
| 1:4 | 326 | 331 |
| 1:2 | 326 | 331 |
| 1:1 | 327 | 331 |
| 2:1 | 327 | 330 |
| 4:1 | 327 | 330 |
| 6:1 | 327 | 329 |
| 8:1 | 327 | 329 |
| 10:1 | 327 | 329 |

**Table S2.** Comparison of the half-life in 40 vol.% DMSO for DhaA with different concentrations of PAH.

| **PAH:DhaA** | ***T*_1/2_ (min)** |
| --- | --- |
| 0:1 | 55±3 |
| 4:1 | 52±2 |
| 8:1 | 30±2 |

**Table S3.** The averaged non-bond binding energy between water molecules and the overall structure of the enzyme DhaA in the absence/presence of PAH.^a^

|  | **ΔE_vdW_ (kcal/mol)** | **ΔE_elec_ (kcal/mol)** | **ΔG_non-bond_ (kcal/mol)** |
| --- | --- | --- | --- |
| DhaA | -208±9 | -6006±144 | -6214±135 |
| DhaA+4PAH | -250±14 | -4733±125 | -4983±135 |

^a^ The data were averaged from the last 40 ns in three independent molecular dynamics (MD) runs. ΔE_vdW_: the van der Waals energy. ΔE_elec_: the electrostatic energy. ΔG_non-bond_: the non-bond binding energy. ΔG_non-bond_ = ΔE_vdW_ + ΔE_elec_.

**Table S4.** The averaged non-bond binding energy between DMSO molecules and the overall structure of the enzyme DhaA in the absence/presence of PAH.^a^

|  | **ΔE_vdW_ (kcal/mol)** | **ΔE_elec_ (kcal/mol)** | **ΔG_non-bond_ (kcal/mol)** |
| --- | --- | --- | --- |
| DhaA | -644±6 | -660±34 | -1304±37 |
| DhaA+4PAH | -611±33 | -352±72 | -963±104 |

^a^ The data were averaged from the last 40 ns in three independent MD runs. ΔE_vdW_: the van der Waals energy. ΔE_elec_: the electrostatic energy. ΔG_non-bond_: the non-bond binding energy. ΔG_non-bond_ = ΔE_vdW_ + ΔE_elec_.

**Table S5.** The averaged non-bond binding energy between water/DMSO molecules and PAH chains.^a^

|  | **ΔE_vdW_ (kcal/mol)** | **ΔE_elec_ (kcal/mol)** | **ΔG_non-bond_ (kcal/mol)** |
| --- | --- | --- | --- |
| PAH-water | 45±7 | -82390±229 | -82345±236 |
| PAH-DMSO | -651±7 | -13779±115 | -14431±109 |

^a^ The data were averaged from the last 40 ns in three independent MD runs. ΔE_vdW_: the van der Waals energy. ΔE_elec_: the electrostatic energy. ΔG_non-bond_: the non-bond binding energy. ΔG_non-bond_ = ΔE_vdW_ + ΔE_elec_.

**Table S6.** The averaged non-bond binding energy between PAH chains and the overall structure of enzyme DhaA.^a^

|  | **ΔE_vdW_ (kcal/mol)** | **ΔE_elec_ (kcal/mol)** | **ΔG_non-bond_ (kcal/mol)** |
| --- | --- | --- | --- |
| DhaA-PAH | -33±5 | -11001±584 | -11035±585 |

^a^ The data were averaged from the last 40 ns in three independent MD runs. ΔE_vdW_: the van der Waals energy. ΔE_elec_: the electrostatic energy. ΔG_non-bond_: the non-bond binding energy. ΔG_non-bond_ = ΔE_vdW_ + ΔE_elec_.





**Figure S1.** The contour fluorescence spectra of DhaA.





**Figure S2.** Fluorescence spectra of DhaA in the presence of different concentrations of PAH in (a) the aqueous buffer and (b) 40 vol.% DMSO solution.





**Figure S3.** The kinematic viscosities of (a) the aqueous buffer and (b) 40 vol.% DMSO solution containing different concentrations of various polymer additives.





**Figure S4.** The catalytic rate of 4-bromomethyl-6,7-dimethoxycoumarin by DhaA at different PAH concentrations in 40 vol.% DMSO as a function of substrate concentration. Error bars represent the standard deviations from three independent experiments. The solid lines were calculated from the Michaelis-Menten equation with the kinetic parameters listed in Table 1.





**Figure S5.** Circular dichroism (CD) spectra of DhaA in the absence/presence of PAH in the aqueous buffer and 40 vol.% DMSO. It should be noted that due to the interference of DMSO to CD signals in the far-UV spectral region, CD data below 220 nm could not be obtained in 40 vol.% DMSO.





**Figure S6.** The residual activity of DhaA at different PAH concentrations in 40 vol.% DMSO at 30 ^o^C as a function of incubation time. Error bars represent the standard deviations from three independent experiments. (a) Relative residual activity of the enzymes as compared to the initial activity of the enzyme in the corresponding solution; (b) the residual activity of the enzymes by defining the initial activity of pure enzyme (no PAH) as 100%.





**Figure S7.** Fluorescence spectra of DhaA with different PAH concentrations in 40 vol.% DMSO solution (a) before and (b) after 90 min of incubation.





**Figure S8.** The catalytic activity of DhaA with/without adding PAH in different concentrations of (a) DMSO, (b) N,N-dimethylformamide (DMF), and (c) ethanol solutions. The figure was redrawn from Figure 2 by defining the activity of enzyme with adding corresponding PAH concentration in aqueous buffer as 100%.





**Figure S9.** Fluorescence spectra of DhaA with/without adding PAH at different DMSO concentrations.





**Figure S10.** Fluorescence spectra of DhaA with/without adding PAH at different DMF concentrations.





**Figure S11.** Fluorescence spectra of DhaA with/without adding PAH at different ethanol concentrations.





**Figure S12.** Fluorescence spectra of DhaA with different polymer additives in (a, b) aqueous buffer and (c, d) 40 vol.% DMSO.





**Figure S13.** (a) Root mean square deviation (RMSD) of the Cα atoms and (b) the time course of radius of gyration (R_g_) analysis of the enzyme in the absence/presence of PAH using MD simulations.





**Figure S14.** The time-averaged total, hydrophobic, and hydrophilic solvent accessible surface area (SASA) of the enzyme in the absence/presence of PAH in 40 vol.% DMSO. The average of SASA was computed based on the last 40 ns of each simulation. Error bars represent the standard deviations from three independent MD simulation runs. Here, SASA referred to the surface area of enzyme, accessible to water molecules and OS molecules calculated using a probe of radius 1.4 Å. The cut off -0.2 to 0.2 was used for hydrophobic and hydrophilic SASA calculations.





**Figure S15.** The number of hydrogen bonds between enzyme DhaA and PAH chains as a function of simulation time.
